# Ethical challenges in mass drug administration for reducing childhood mortality: a qualitative study

**DOI:** 10.1186/s40249-022-01023-6

**Published:** 2022-09-16

**Authors:** Ahmed Alasmar, Alex C. Kong, Anthony D. So, Matthew DeCamp

**Affiliations:** 1 Center for Bioethics and Humanities, University of Colorado Anschutz Medical Campus, Aurora, USA; 2 Innovation+Design Enabling Access (IDEA) Initiative, Department of International Health, Johns Hopkins Bloomberg School of Public Health, Johns Hopkins University, Baltimore, USA; 3 Division of General Internal Medicine and Center for Bioethics and Humanities, Department of Medicine, University of Colorado School of Medicine, University of Colorado Anschutz Medical Campus, 13080 E. 19th Ave, Mail Stop B137, Aurora, CO 80045 USA

**Keywords:** Antimicrobial resistance, Decolonizing global health, Mass drug administration, Child mortality, Ethics, Global health equity

## Abstract

**Background:**

Mass drug administration (MDA) of medications to entire at-risk communities or populations has shown promise in the control and elimination of global infectious diseases. MDA of the broad-spectrum antibiotic azithromycin has demonstrated the potential to reduce childhood mortality in children at risk of premature death in some global settings. However, MDA of antibiotics raises complex ethical challenges, including weighing near-term benefits against longer-term risks—particularly the development of antimicrobial resistance that could diminish antibiotic effectiveness for current or future generations. The aim of this study was to understand how key actors involved in MDA perceive the ethical challenges of MDA.

**Methods:**

We conducted 35 semi-structured interviews from December 2020–February 2022 with investigators, funders, bioethicists, research ethics committee members, industry representatives, and others from both high-income countries (HICs) and low- and middle-income countries (LMICs). Interview participants were identified via one of seven MDA studies purposively chosen to represent diversity in terms of use of the antibiotic azithromycin; use of a primary mortality endpoint; and whether the study occurred in a high child mortality country. Data were analyzed using constructivist grounded theory methodology.

**Results:**

The most frequently discussed ethical challenges related to meaningful community engagement, how to weigh risks and benefits, and the need to target MDA We developed a concept map of how participants considered ethical issues in MDA for child mortality; it emphasizes MDA’s place alongside other public health interventions, empowerment, and equity. Concerns over an ethical double standard in weighing risks and benefits emerged as a unifying theme, albeit one that participants interpreted in radically different ways. Some thought MDA for reducing child mortality was ethically obligatory; others suggested it was impermissible.

**Conclusions:**

Ethical challenges raised by MDA of antibiotics for childhood mortality—which span socio-cultural issues, the environment, and effects on future generations—require consideration beyond traditional clinical trial review. The appropriate role of MDA also requires attention to concerns over ethical double standards and power dynamics in global health that affect how we view antibiotic use in HICs versus LMICs. Our findings suggest the need to develop additional, comprehensive guidance on managing ethical challenges in MDA.

**Graphical Abstract:**

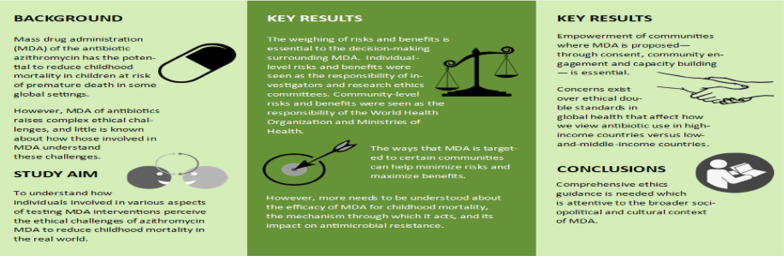

## Background

Mass drug administration (MDA), i.e., empirically administering drugs to an entire at-risk community or population, appears to be a promising global health intervention [1, 2]. MDA has been used to treat trachoma (a bacterial cause of blindness affecting nearly 2 million people annually) [3, 4], and MDA programs are often used to control other neglected tropical diseases [5–8]. MDA programs have been applied in efforts to reduce the incidence of lymphatic filariasis, onchocerciasis, schistosomiasis, and soil-transmitted helminth infection [9–12] (though a recent meta-analysis found little evidence of deworming’s positive effects on height, anemia, cognition, school performance, or mortality[13]).

Recently, MDA to reduce childhood mortality has received considerable attention, spurred by a perception that it is safe, effective, and inexpensive [14], and by results from the Macrolides Oraux pour Réduire les Décès avec un Oeil sur la Résistance (MORDOR) trial of the broad-spectrum antibiotic azithromycin given twice yearly to children under five in sub-Saharan Africa. MORDOR showed a statistically significant reduction in mortality overall, but this was only significant in one of three study sites [15, 16]. Rather than target a specific disease, MORDOR investigated further the unintended mortality benefits seen in prior azithromycin trials for trachoma [17]. The World Health Organization (WHO) has since issued recommendations on the use of azithromycin MDA in some resource-limited settings [18].

However, azithromycin MDA for childhood mortality raises complex ethical questions [19]. These include how to weigh near-term gains against longer-term consequences (e.g., decreased childhood mortality now versus risk of antibiotic resistance later) [20, 21] and how to distribute fairly benefits and risks between individuals, communities, and whole societies [22]. MORDOR did find an increase in azithromycin-resistant organisms in nasopharyngeal and gastrointestinal samples following the MDA [23, 24]. The mechanism of MDA with azithromycin to reduce mortality is not understood, and all populations may not benefit equally [25]. Evidence of even near-term benefits is conflicting [26, 27], and potential longer-term risks, such as increased obesity risk due to antibiotic-induced changes in the gut microbiome [28] or diminished clinical effectiveness of frontline antibiotics due to antimicrobial resistance (AMR) [29], have not been fully assessed.

Bioethics research on MDA with antibiotics—especially when applied to reduce childhood mortality—is limited. Although ethical issues have been explored in other contexts, such as MDA for seasonal malaria chemoprophylaxis [30] or soil-transmitted helminths [31], MDA for childhood mortality is arguably distinct. To our knowledge, no empirical studies describe how critical decision-makers think about ethical concerns in MDA of antibiotics. Understanding their perspectives can yield new insights into the ethics of MDA generally and inform future guidance development. In its 2020 guidelines, the WHO indicated the need to revisit its guidance on the use of azithromycin MDA in 2–3 years. Without the benefit of such perspectives, some ethically relevant factors may be neglected, leading to future unanticipated, but preventable, harms [32]. To fill this knowledge gap, we sought to understand how individuals involved in various aspects of testing MDA interventions perceive the ethical challenges of MDA in the real world.

## Methods

Given the absence of existing data on this topic, we chose to conduct a qualitative, empirical bioethics study using semi-structured interviews from 30 December 2020 –15 February 2022.

### Sample

To select interviewees, first, we reviewed published literature and clinical trial registries (clinicaltrials.gov and the WHO International Clinical Trials Registry, https://www.who.int/clinical-trials-registry-platform) to identify ongoing and completed MDA studies. Recognizing no standard definition of what counts as an MDA study (such as in terms of number of participants enrolled or how narrowly or broadly the drug is used in a community), we sought to include studies that could inform MDA as a potential public health intervention. We then used a purposive sampling approach [33, 34] to obtain a diversity sample across three criteria: (1) whether or not azithromycin was used, (2) whether or not the trial had a mortality endpoint, and (3) whether the country where the trial occurred could be classified as a high mortality country (infant mortality > 60 per 1000 live births or under five mortality > 80 per 1000 live births), reflecting WHO guidance for MDA azithromycin to reduce child mortality [18].

Second, using these criteria, we identified seven high priority studies. Individuals from one study did not respond to recruitment emails; we replaced this study with an additional one. The final list of trials from which we recruited is presented in Table 1.

**Table 1:** Descriptions of clinical trials included in our study and from which interview participants were recruited

TRIAL	Description	Location	High child mortality country	Azithromycin used in study	Mortality outcome
Mortality Reduction After Oral Azithromycin: Mortality Study (MORDOR)	A cluster-randomized trial with 3 sites comparing communities where children aged 1–59 months receive biannual oral azithromycin ("Azithromycin" arm) for two years, to communities where the children receive biannual oral placebo ("Control" arm) for two years in order to assess childhood mortality	Malawi Niger Tanzania	Yes (Niger)	Yes	Yes (Primary)
Effects of Mass Drug Administration of Azithromycin on Mortality and Other Outcomes Among 1–11 Month Old Infants in Mali (LAKANA)	A cluster-randomized trial in Mali to assess the impact on mortality and other health outcomes of quarterly and biannual azithromycin mass drug administration (MDA) when delivered to 1–11-month old infants in a high-mortality setting where malaria is holoendemic but there is also a functioning seasonal malaria chemoprevention (SMC) program in place	Mali	Yes	Yes	Yes (Primary)
Early Life Interventions for Childhood Growth and Development in Tanzania (ELICIT)	A randomized, 2 × 2 factorial, double-blind, placebo-controlled trial in the area around Haydom, Tanzania. Mother–child dyads were enrolled by age 14 days, randomized to 1 of 4 treatment arms, and followed with monthly home visits and every 3-month anthropometry assessments through 18 months. Primary outcome was length-for-age z-score (LAZ) at 18 months in the modified intention-to-treat group	Tanzania	No	Yes	Yes (Secondary)
Azithromycin—Ivermectin Mass Drug Administration for Skin Disease (AIM-Skin)	An open-label prospective community intervention trial to assess the impact of community mass treatment with azithromycin for yaws and ivermectin for scabies, on non-yaws bacterial skin infections. Communities were randomized to receive standard treatment for both yaws and scabies either in parallel (site 1) or in sequence (site 2). Primary outcome was the difference in the change in prevalence of impetigo between baseline and 12-months between the parallel and the sequential treatment arms	Solomon Islands	No	Yes	No
A Trial of Seasonal Malaria Chemoprevention Plus Azithromycin in African Children (SMCAZ)	A randomized, placebo-controlled trial in the Hounde district of Burkina Faso and the Bougouni district of Mali. Children aged 3–59 months were randomized to receive four cycles of either SMC and azithromycin or SMC and placebo at monthly intervals during peak malaria transmission season. Primary outcome was the incidence of the combination of death or hospital admission for at least 24 h, not due to trauma or elective surgery during the intervention period	Burkina Faso Mali	Yes	Yes	Yes (Primary)
Nutritional Support for Lactating Women and Azithromycin to Infants—Mumta Lactating Women Trial (MumtaLW)	A community-based, randomized control trial in peri-urban settings of Karachi, Pakistan to study the impact of lipid-based nutritional supplement for pregnant and lactating women (LW) and single prophylaxis dose of azithromycin for infants on growth (length velocity) of infants over the period of six months since birth compared to current standard of care. LW and her infant are enrolled in the trial within 168 h of birth and will be randomized to one of three study arms: standard of care (control), nutritional supplement only, or nutritional supplement plus azithromycin	Pakistan	No	Yes	No
The Effect of Routine Antibiotic Use in the Outpatient Treatment of Severely Malnourished Children Without Complications	A randomized, placebo-controlled trial to compare routine antibiotic prescription vs no routine antibiotic prescription in the management of uncomplicated cases of severe acute malnutrition treated in a community in Niger in terms of nutritional recovery. Children aged 6–59 months received either routine amoxicillin prescription for 7 days (80 mg/kg/day) or placebo. Primary outcome was proportion of children discharged from nutritional program as recovered	Niger	Yes	No	Yes (Secondary)

Third, we started by recruiting clinical investigators and then relied upon chain referral sampling to recruit other participants. For instance, during interviews, we asked investigators to connect us to institutional review board (IRB) or research ethics committee (REC) members (hereafter referred to under the single acronym, REC), data safety and monitoring committee (DSMC) members, ministry of health officials, funders, drug company representatives, community members where MDA trials have occurred, bioethicists, and individuals from intergovernmental organizations. We intentionally sought balance among individuals from high-income countries (HICs) and low- and middle-income countries (LMICs).

### Data collection

We designed an interview guide based on existing MDA ethics literature, ethical issues in global health research, and the research team’s experience in conducting qualitative interviews and expertise in global health policy. The interview guide aimed to gain insight into participants’ personal experiences with ethical issues in MDA. Consistent with qualitative methods, the guide was modified over time to incorporate insights learned from prior interviews. One member of the study team (MD) conducted 35 semi-structured interviews between 30 December 2020 and 15 February 2022. Each lasted approximately 1 h. All were transcribed verbatim.

### Data analysis

Data analysis utilized grounded theory—a methodology ideal for creating models or maps of the relationships between concepts [35]. Specifically, we applied Charmaz’s constructivist version [36] of classical grounded theory which acknowledges that the analysis is tied to particularities of time, place, and the researchers’ own vantage points.

First, immediately after each interview, field notes and memos were written to aid analysis. Throughout data collection, transcripts (20 out of 35) were also shared and reviewed with all four members of the study team in order to identify patterns emerging from the data and themes that warranted deeper exploration in subsequent interviews. To promote objectivity, two team members (ACK and ADS) not involved with data collection reviewed transcripts independently and led these sessions.

Second, two authors (AA and MD) began “open coding.” We reviewed six transcripts chosen for diversity of trials and stakeholder type. Remaining close to the text [37], a preliminary set of descriptive codes was developed.

Transcripts were uploaded into ATLAS.ti (version 9, Windows, Scientific Software Development GmbH, Berlin, Germany) to assist with data management. Transcripts used in open coding were re-coded, and constant comparative techniques [38] were applied to add and clarify categories, codes, and the relationships between them (“axial” coding). Five transcripts were coded by both coders (MD and AA) who met to review each to ensure coding reliability.

In the final stage of analysis, two study team members (MD and AA) met multiple times to review all the data, draw and re-draw diagrams of the relationships between concepts, and identify a “core” category (or main theme) of our findings. Our analysis was informed further by discussions with the research team (ACK and ADS) as well as five, 90-min expert consultation sessions held virtually as part of the overall funded project. To ensure rigor, we employed standard qualitative techniques, such as reflexivity (openly acknowledging our own potential biases regarding MDA), triangulation between different stakeholders, and member checking (by sharing our findings back with research participants).

## Results

### Characteristics of participants

Demographic data for interview participants are in Table 2. Our final sample included 35 interviews (one interview was a group interview with a national research ethics committee where MDA trials had occurred, who preferred this format). Because MDA in global health using antibiotics is a relatively small field, we intentionally report minimal demographic data to protect participant anonymity.

**Table 2:** Demographic characteristics of interviewed participants

Characteristic	*n* = 34^a^
Age, years	
30–39	4
40–49	9
50–59	5
60–69	9
70–79	6
80 +	1
Gender	
Female	8
Area of expertise	
Infectious disease	10
Public/Community health	9
General medicine	7
Bioethics	3
Ophthalmology	3
Nutrition	2
Nationality	
USA	13
UK	5
Uganda	2
Tanzania	2
Nigeria	2
Pakistan	2
France	1
Norway	1
Finland	1
Sweden	1
Burkina Faso	1
Ethiopia	1
Bangladesh	1
Australia	1
Stakeholder type^b^	
Investigator	17
Funder	7
Intergovernmental organization	4
Bioethicist	4
Institutional Review Board or Research Ethics Committee member	3
Data Safety and Monitoring Committee member	3
Community health expert	2
Local community member	2
Government official	1
Industry	1

^a^34/35 Interviewees are represented in this table. One interview with a group of individuals from a national research ethics committee where mass drug administration has occurred is not included

^b^For stakeholder type, *n* > 35 because some participants could be classified as more than one stakeholder type

### Thematic codes

Table 3 displays the ten most frequently appearing codes from our interviews. Although coding frequency alone is not necessarily indicative of importance, it provides a first glimpse into interview content.

**Table 3:** Codes which appeared most frequently in interviews

Code group	Codes	Instances of coding
Community engagement	Need meaningful community engagement	56
	Essential role (including community health workers)	21
	MDA’s interaction with health system	19
	Need for capacity building	18
Risks and benefits	Weighing risks and benefits	41
	Ethical double standard	26
	Indirect vs. direct benefit	22
Targeting and implementation	Need to know mechanism before implementation	18
	Bridging gap between research and implementation	15
	Baseline AMR	15

*MDA* mass drug administration, *AMR *antimicrobial resistance

The most frequently appearing codes related to community engagement. All interviewees felt that meaningful involvement of communities in the decision-making surrounding MDA was essential. As one participant said:

…because it’s a community, you need to engage these communities…you have to pass through the community leaders…if you, say, in district level, you get support, perhaps you have people joining your team… and these people will help you to mobilize these people to come and accept this mass drug. [REC member, LMIC]

However, another participant saw the importance of engagement in part through a concern over its absence or authenticity:

…over the last 20 years, we've seen at least language put forward about being more inclusive in decision-making of the communities in which these interventions are being proposed. But in some ways, that's almost more dangerous, if it's not really happening…we've created this false sense of self-assurance…but we're really not enabling them with the tools and the information and everything that they need to really, truly make well-informed, independent decisions… does Niger want to do MDA in Niger? [Investigator & DSMC member, HIC]

A second group of codes in Table 3 relates to risks and benefits. As we describe more below, participants had differing opinions about which risks/benefits mattered, how to weigh them, and who should be responsible. One stated:

…is it acceptable for some districts in the world where more than 80 kids per thousand die before their fifth birthday? If it's not acceptable, what can you do about it? Well you could improve vaccination coverage. That's pretty good in most of those West African countries...You know, you can improve the standard of living, improve water and sanitation. That's expensive and a long-term project. So in the short term…what can you do? Well, I think giving twice yearly azithromycin will reduce under five mortality…So is it ethical not to do that? You know…in those communities where they're not using macrolides between rounds of mass treatment, we don't think selection of resistance is a big problem. [Investigator, HIC]

Others saw this calculus differently:

But always my concern has been, since we have been using this medicine for trachoma at a larger scale, especially in Ethiopia…I have slight worry that it may be a reason for some resistance…So I don't encourage mass drug administration as such, honestly speaking. [Funder & Investigator, LMIC]

A third code group relates to the targeting and implementation of MDA. Participants expressed a need to understand the mechanism before MDA is widely implemented. For instance, a research ethics committee in a country where MDA has taken place thought that the use of “preventive” antibiotics, while not impermissible, deserved special scrutiny when compared to using antibiotics as a “cure” for a specific illness. One investigator said frankly:

To date, I don't think anybody has been able to explain a plausible causal mechanism for the results [of azithromycin MDA for mortality]…And once you implement something like that outside of the confines of the study, it's extremely difficult to stop doing it. [Investigator, HIC]

Another recommended that the global health community ought not to stop doing research after only a few studies showed positive results:

So if you look at the science, do we have enough evidence for the mass drug administration?... perhaps investing more in investigating the failures...If you take azithromycin this number of rounds, prevalence should go down, but we see it-- the prevalence goes down, then one, two years down the road, the prevalence is up again. So what are those other factors that are driving prevalence that need to be looked into besides the mass drug administration? [Funder, LMIC]

### Concept map

**Fig. 1:**
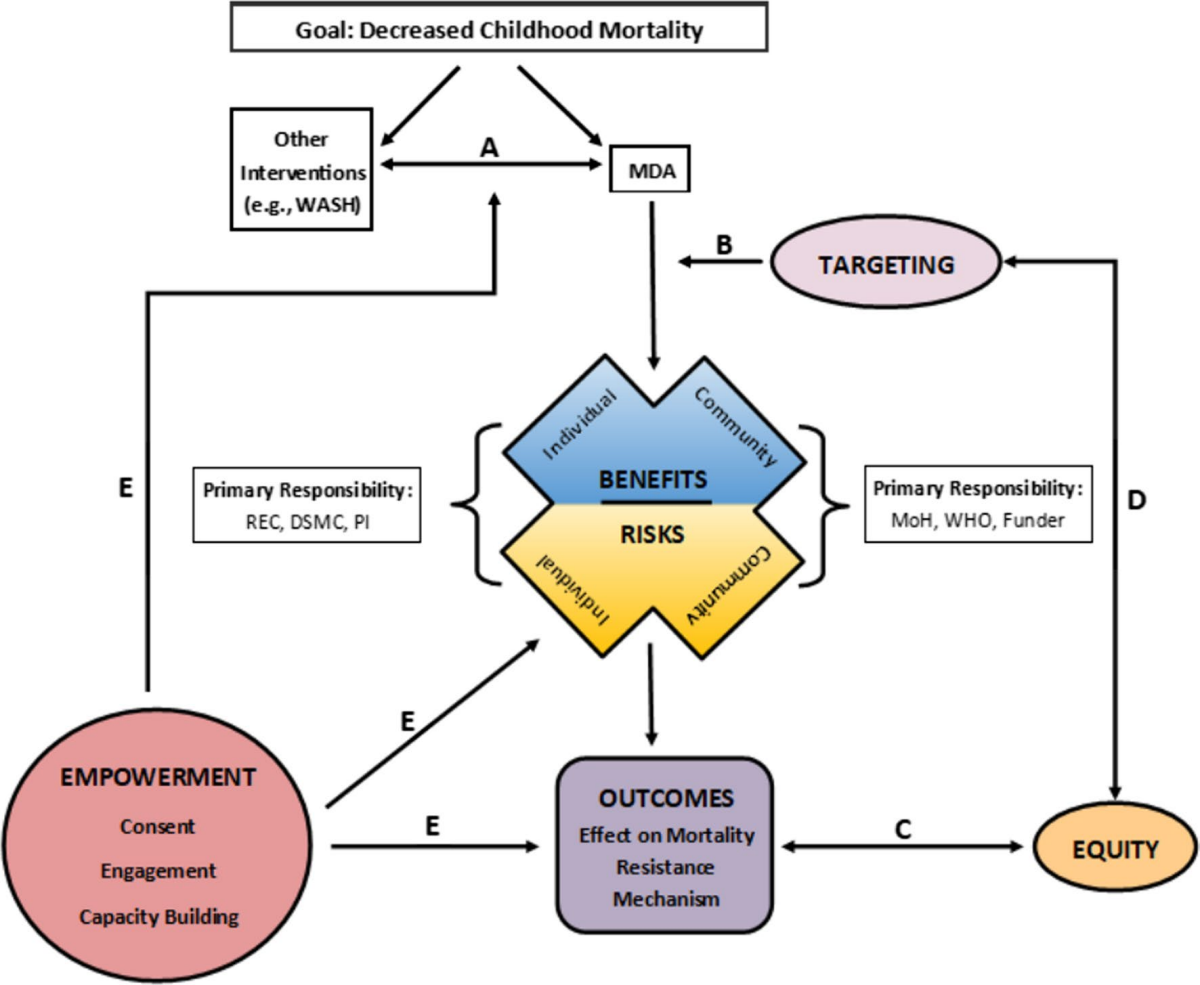
Concept map developed from interview coding—arrows with letters are referenced in text. *DSMC* data safety and monitoring committee, *MDA* mass drug administration, *MoH* ministry of health, *REC *research ethics committee, *PI* principal investigator, *WASH* water, sanitation, and hygiene, *WHO* World Health Organization

From our codes, we developed the primary product of our analysis – a concept map (Fig. 1). This map is our interpretation of the relationships between the most important themes, as learned from our participants.

The map acknowledges first (Fig. 1, top left), that a key question is, “Why MDA?” in comparison to other public health interventions (e.g., WASH, or water, sanitation, and hygiene programs), or pursuing both simultaneously (Fig. 1, arrow A). We found no consensus answer to this “why” question. We heard concerns that MDA would be used instead of other proven interventions, as a pejorative “magic bullet”:

…there’s been this huge tension in global health about the magic bullet compared to…working together for the common good. I mean, they’re not mutually exclusive, but we have a long history of being drawn to ‘magic bullets’…working with communities and helping them to improve their health just haven't received the degree of support that they should have… [Community Health Expert, HIC]

…you find most partners but also most donors tend to focus more on the biomedical, which is the surgery and the antibiotics because…they are easy to measure, easy to count...sometimes behaviors take long to change, [are] not easy to count… [Funder, LMIC]

Others emphasized that MDA “versus” other interventions was a false dichotomy:

…these are other programs that could help the diseases, one, and also help mortality…without the community ending up in a position whereby they would require a mass distribution of drugs …many times these [MDA] programs are seen as standalone programs where from a public health point of view, it should be in conjunction with so many other interventions**.** [Investigator, LMIC]

Why are you spending all this money on this and why aren't you doing water and sanitation interventions?...the only answer is, well, of course, they're not mutually exclusive…like I'm doing MDA because it's easy…when what you should be doing is addressing these underlying structural problems. To which I think the answer is well we should do both…it's not fair to let people go blind just because it's going to take 50 years for there to be sufficient economic development…So the thing to do is to pursue MDA whilst pursuing the economic development, which will hopefully make MDA go away. [Investigator, HIC]

Weighing risks and benefits at the individual and community levels is central in the map (Fig. 1, center). Participants discussed direct risks to individuals who receive MDA—including near-term risks, such as adverse drug reactions or the physical risk of choking on tablets—as well as longer-term ones, such as the risk of a future drug-resistant infection. Only a few participants, without prompting, noted possible health but non-AMR risks (e.g., changes to an individual’s microbiome or longer-term health risks from early antibiotic exposure, such as obesity), suggesting these were not high on interviewees’ minds. While a reduced likelihood of mortality was perhaps the most obvious direct individual benefit, others wondered about other health outcomes (e.g., improved cognition or development) that might come from MDA of azithromycin.

Participants saw possible community-level benefits from MDA research generally. These could include a reduction in diarrheal or respiratory disease burden, or improvements to the local health system as a result of running the MDA study (while acknowledging that such a benefit is not unique to MDA studies). For one participant, such benefits were just as important as any benefit on mortality:

So at the very heart of where I see its value or its potential value is not in the intervention itself, but in the platform… the idea of a community-based intervention that's often led by community volunteers from the communities in which the intervention is being targeted and getting out of the traditional siloed structures of the health system or the education system offers incredible opportunity to reach the less reached… there's a huge amount of unmeasured indirect benefits…in terms of contact with some form of a health care worker or even a volunteer health care worker… [Investigator, HIC]

Community-level risks—particularly development of AMR—were challenging for participants to understand or weigh. Increasing AMR could negatively impact members of the community who did not directly benefit from the MDA (i.e., who did not receive the drug). While participants saw AMR as a major challenge, disagreement existed over how to weigh this risk. Some wondered whether the total volume of azithromycin used in mortality MDA would actually be far less than in already accepted programs, such as for trachoma. Demonstrating the diversity of views are the following:

No one can tell me how much resistance is too much. [Funder, HIC]

…resistance is a funny thing. Because it doesn't necessarily, it's a population issue, more than an individual issue. [Investigator, HIC]

So if drug resistance at a community level increases by 5%, how do you measure that against a reduction of 2% mortality? What equation are you going to write to measure those? We kind of spitball and say, ‘Yeah, that sounds good enough.’ …we can tolerate a little bit of antimicrobial resistance and we can't get funding to really monitor it anyway, so let's just close our eyes and go for it… But I don't think we have even ethically grounded clarity on how to balance or how to decide those things. [Bioethicist & DSMC member, HIC]

Resistance from MDA of azithro for mortality is such a drop in the bucket compared to agricultural use or compared to even trachoma use of azithro. [Bioethicist & DSMC member, HIC]

And the risk is, using azithromycin as we did, we found that we create a lot of resistance to these antibiotics… [Investigator, LMIC]

One participant implored clarifying the absolute and relative risks associated with AMR and mortality (e.g., older children may have lower relative risk of mortality, but because there are more of them in the population, the net effect on mortality from MDA could be greater):

…there've been some interesting presentations about absolute and relative risk. And so there are more children between one and five than less than one…on an absolute mortality benefit -- it'd be better to include more children, but the relative risks you know, were less in the older children. [Industry representative, HIC]

In another instance, a participant recommended that understanding community risks of AMR required monitoring resistant clinical infections at nearby hospitals (while also noting that there were insufficient resources for this):

So the real litmus test would be if we could be in health facilities and look at critically ill people and then look if they have resistant clones that share a genetic marker with those exposed kids who now have resistance…And you could then think that because you have given this intervention here, now, at the health facility, you start getting sick people. And this is really what we wanted to do and [what] WHO wanted us to do...[Investigator, HIC]

Targeting moderates benefits and risks, because how MDA is targeted (i.e., to which communities MDA is given) influences the extent of risks and benefits (Fig. 1, arrow B). To illustrate, targeting based on the background antibiotic use in the community can influence the benefit/risk calculation—the risk of AMR might be less if background antibiotic use is low, as selective pressure is less. In general, we found hesitancy regarding MDA in settings where azithromycin has been used widely, and where azithromycin is used to treat endemic diseases, such as drug-resistant typhoid (where development of resistance has greater consequences).

…the word that I like here is optimization… We're not chucking antibiotic by the bucket load with no end in sight like they are in agriculture. It is targeted to the people that need it. There is a felt benefit, there is a need and once that need is over, you stop. And that's an optimal use, even though it's hundreds of millions of doses. [Funder, HIC]

How best to target MDA was a critical point of discussion among our participants. Participants saw the need to understand MDA’s mechanism, background antibiotic use, and baseline AMR in a community before implementation in order to maximize benefits while minimizing unnecessary risks:

The biggest evidence gap is, we do not know its mechanism…[another] of the gaps was, listen, we want to know what the effect on AMR is, but we don't even know what the AMR situation is right now in those countries…the WHO even in the high mortality settings where they have recommended azithromycin, you must have seen the little placeholder… while you are doing this, you also need to know what's going on [as far as AMR]. [Investigator & Expert consultant in WHO guideline development, LMIC]

Through our interviews, we also gained a general sense of the responsibilities of various stakeholders in weighing and managing benefits and risks (Fig. 1, boxes to either side of the benefits and risks). Based on our review of how participants identified responsibility in interviews, individual benefits and risks were largely considered to be the responsibility of RECs, DSMCs, and investigators, while community benefits and risks were seen more as a responsibility of ministries of health, the WHO, and funders. When asked who should be responsible for weighing longer-term, community-level risks and benefits, one participant said the following:

That's an easy answer but it's a difficult answer too because, of course it should be the people that have invested with this responsibility, your health care people that are responsible for the community starting from the Ministry of Health, you know. But unfortunately, sometimes they got problems. So…within a health system that trying to study long-term implications of an intervention may not necessarily be the number one thing on someone's to do list. [Investigator, LMIC]

Equity plays a key role in the map because who benefits from an MDA, and how much, are issues of equity, and outcomes should be influenced by concern for equity (Fig. 1, arrow C). Moreover, equity intersects targeting (Fig. 1, arrow D), because how MDA is targeted will affect how the benefits and risks of MDA are distributed in a community, and equity concerns should affect how MDA is targeted. Some participants saw MDA as inherently equity promoting:

If you're looking at, for example, schistosomiasis, which is school-based programming, as children in some countries get older, the female students drop out of school. So then, obviously, you're not reaching them as much as you would be when they were younger. But for house-to-house, it's actually quite an equitable distribution when you're looking at access to healthcare and interventions…So I think there are ethical issues around mass drug administration as an intervention, but…there's a lot of equity in the fact that we like to think that we're treating people at the end of the road… [Investigator, HIC]

However, for others, equity was not to be assumed; even MDA requires attention to fair access among all populations:

Then actually last year we had a challenge between the border districts between Uganda and Kenya that in that stretch of the eastern border of Uganda and the western border of Kenya, there are migratory populations. These are nomadic people. They are moving for pasture, for their cows… the borders don't matter…Yet when you look at the way the azithromycin distribution has been organized, it's basically around the borders…So we innovated. Started doing cross-country joint mass drug administration so that the drug administration is synchronized…[otherwise] you're having a huge number of people who are basically missing out on the mass drug administration. [Funder, LMIC]

A final critical concept is that of empowerment. Empowerment influences the decision to use MDA in the first place, affects how benefits and risks are experienced (and who weighs them), and influences the outcomes of a study (particularly, the community acceptability of MDA) (Fig. 1, E arrows). Empowerment includes consent, community engagement, and capacity building; it reflects the idea that community members should be the primary decision-makers regarding MDA in their communities.

At the individual level, consent was seen as a way to empower people who participate in MDA; in practice, we heard concerns that HIC RECs were not obviously committed to empowerment:

If I've got to decide the way things were run, I would say make the consent process around the individual, not about your university...I think it should be to do the best we can to communicate to that individual that some of the kids are going to be treated. Some of the kids are not going to be treated…etc. [Investigator, HIC]

Along with being ethically obligatory, empowering communities is practically necessary for MDA. Community health workers (CHWs) play an essential role in successful MDA. Not only do CHWs often serve as the main contact with participants (with responsibilities including drug delivery, fielding questions, and recording adverse events), but they also have an ongoing role once foreign investigators leave. One community health expert said:

The integrated community health worker program has to exist before you bring in the azithromycin. But of course…people argue that things like azithromycin or family planning or immunizations, they can serve as an entry point for building a stronger, more integrated permanent healthcare program…But to my way of thinking, we need a much stronger emphasis on helping countries realize the value of having a strong community health worker program. [Community heath expert, HIC]

Finally, participants identified meaningful community engagement and capacity building as ways to cultivate real ownership of MDA by the community—including the decision about whether to use MDA in the first place:

…country ownership really means that countries have to drive programs and that our money should follow that, and I don't think it does always. We need to give national governments the freedom to be able to follow the agendas that they want to follow rather than sort of semi-listen, but then create sort of almost divisive lock frames and divisive deliverables that actually detract and distract from what's important. So I think that funders have to really follow their words and promise of country ownership…And that's a risk. Any funder is always concerned about the risk around financial governance. But then they need to build capacity around it rather than micromanage it. [Investigator, HIC]

In closing, we note that the figure displays an iterative process. Following the arrows shows how what is learned from each MDA study adds to the evidence base and can influence subsequent guideline development:

So whatever these guidelines are evolving. These are guidelines based on studying evidence in settings…So when these guidelines were developed, it was based on studying evidence in that particular time period…it should be continuously evolving based on new evidence. [Investigator, LMIC]

### Ethical double standard: the unifying theme

One concept—the “ethical double standard”—emerged as a unifying theme. The ethical double standard surrounded how MDA is viewed from the vantage point of people in HICs compared to LMICs. It was multidimensional, and participants had very different ways of thinking about it. For some, the double standard is inherent to MDA because extremely high childhood mortality is a problem only in LMICs and is ultimately a socio-economic problem; the need for MDA creates a double standard via an intervention in LMICs that would be unacceptable in HICs:

…look, we would never recommend MDA with, for instance, azithromycin, I don't know, in New York, right?…it's not necessarily the risk of AMR that pushes us to not do it. Aside from the fact that child mortality is incomparable to the countries where you introduced it, the main reason is that there are alternatives… [Bioethicist & Expert consultant in WHO guideline development, HIC].

It's not the fact that you have pneumonia that kills you. It's not the malaria. It's poverty, it's poverty that kills these kids. And we don't have an anti-poverty pill or an anti-poverty vaccine. [Funder, HIC]

Because they are in underdeveloped contexts -- ‘OK, so if we're not going to have enough research to be able to tackle these things properly and we may not have drugs to treat them effectively, then we make do with what we have.’ And what we have now is one option is to just go and treat the community with a particular drug. It will do the trick, but I don't think that if you tried that in Paris, I don't think it will be that acceptable to have that done. [Investigator, LMIC]

Others, even if they agreed that MDA may not be the optimal solution to reducing childhood mortality, described the ethical double standard as reluctance to use antibiotics in LMICs when, in HICs, antibiotics are used in greater amounts and for comparatively minor or inappropriate purposes (e.g., as an acne treatment or inappropriately for a viral illness):

So, you know, it's fine and good sitting in [HIC city] to say we shouldn't treat kids because there might be resistance. But when child mortality is as high as it is in Niger, the risk of not doing something is that, you know, five, six, percent of the kids are going to die…It would be unethical not to accept some level of risk based on our worries about these potential effects that are down the road...many people, I think, are worried about the emergence of resistance. But you know, they have no problem going to their own pediatrician and demanding antibiotics for every little ear pain. And yet they don't want kids in Niger getting azithromycin because it might drive resistance. And that, I think, is a real ethical issue. [Investigator, HIC]

Related to our map, participants’ views of the ethical double standard influenced how they weighed risks and benefits. Is the level of risk that HICs consider acceptable different from what might be acceptable in LMIC settings where the potential benefit is much greater? If so, how do we navigate this, and who should be making decisions? One participant stated:

I think when we compare studies in low-income countries to studies in high-income countries, then we also need to adjust what we find an acceptable risk…when we talk about ethics -- that's almost the toughest part because I really struggle with deciding who ought to define that, right? So clearly, in a country where child mortality is 25 times higher than in the country where I live, an acceptable risk for doing something about that is higher than it's here, right?...But am I the right person to do that? Is that a national decision? Is that something the WHO can recommend?...And for me to have an opinion on what an acceptable ratio of risk to benefit in a low-income country for a three-year-old ought to be is so alien to my own world and my own experiences …but it almost feels wrong to me to even have an opinion on that...[Bioethicist & Expert consultant in WHO guideline development, HIC]

Finally, some believed the double standard to be reflective of an ethnocentrism that assigned greater value to people in HIC’s than those from LMIC’s.

I do think azithromycin is an intervention that is used by people in the West, especially for kids, and this is also the place where the worry about drug resistance is growing in many areas…And I have to say that there is a little bit of ethnocentrism and in some ways indirect racism about, well, ‘if you people in Africa are just going to give it out to everybody then we're not going to be able to treat people anywhere with this antibiotic.’ Which may in the end be true, but the question is what's the value of a human life? … We have similar thematic arguments with genetically modified mosquitoes for malaria control. Yeah, you got people in Birkenstocks in the West say don't do it, you could have franken-mosquitos and then it's going to destroy the world, it's going to be all your fault. You have people in places like Burkina Faso who are very, very smart, very skilled who are going to say well wait, we don't have any tools, our kids are dying of malaria. So—the power dynamic between the West and the north and the south here is not trivial. [Funder, HIC]

The diversity of opinions regarding this ethical double standard that came up in our interviews speaks to its complexity. What is agreed upon, however, is that we will have to reckon with these opinions in order to understand the ethics of MDA.

## Discussion

We have conducted, to our knowledge, the first empirical study of stakeholders’ perceptions of ethical issues in MDA of azithromycin for the sake of reducing child mortality. In several ways, our findings offer empirical support for prior theoretical scholarship about the ethics of MDA. Obtaining truly informed consent at the individual and community level, for example, is a widely accepted ethical obligation in international research [39] and one that our participants recognized [18].

Of central concern was the ethical challenge of weighing benefits and risks. This weighing occurs along multiple dimensions: time, as both short-term risks (e.g., adverse drug events) and longer-term risks (e.g., changes to the microbiome or environment) are evident [18]; identity, as in the challenge of weighing the value of lives saved now versus lives saved later [21]; AMR and its clinical significance [18, 20–22]; and how to manage risks to children as a research population requiring special protections [19]. Our participants endorsed the obligation to monitor for AMR, even though there was uncertainty regarding the best way (e.g., comparing phenotypic methods of detecting AMR, which are time consuming and difficult in some settings, to genotypic methods, which may be faster but of uncertain clinical significance [40]).

Our participants noted that, although MDA could promote equity by reducing the significant, disparate risk of child mortality, this is not a given. Existing WHO MDA guidelines recognize that equity is not guaranteed if the MDA distribution mechanism misses rural, migratory, or other hard-to-reach populations, or is used in places where it is not effective [18]. Ethically, concern for equity begs us to ask “who” is included (or not) in MDA. Answering this question can also be critical for the success of MDA, as inadequate coverage can hinder its effectiveness.

Lastly, our participants endorsed the need, if MDA of antibiotics for childhood mortality is used, to ensure it is “targeted.” Targeting MDA to those populations most likely to benefit and least likely to experience risks could be an important way of managing the ethical and health risks. Indeed ongoing MDA studies explore targeting to different subpopulations [22, 41].

Our study also enriches our understanding of known ethical challenges and uncovered new ones. For instance, participants suggested concrete ways for targeting MDA. Aggregate child mortality and capacity for risk surveillance are not the only considerations for MDA planning. Other factors that must be considered include (i) baseline antibiotic access, use, and resistance, specific to the antibiotic chosen for MDA, accounting for local regulation of antibiotics; and (ii) awareness of competing clinical conditions for which the antibiotic used in MDA is critically important (e.g., in settings where *Salmonella enterica *Typhi is endemic and azithromycin is a crucial therapeutic). These insights generate hypotheses for future research on targeting MDA.

We came to understand the roles and responsibilities of different stakeholders in managing MDA ethics in four unique ways. First, given the complex nature of ethical tradeoffs involved in MDA, participants placed high value on the WHO in creating guidance for individual countries and managing the complex global risks of MDA (e.g., AMR). This is not to say that all participants agreed with the current WHO guidance on MDA; some thought it too permissive, and others, too restrictive. Nevertheless, all agreed that WHO guidance plays a critical role.

Second, the importance of truly empowering local decision-making—via local investigators, RECs, ministries of health, and so on—was seen as crucial to ethical decision-making around MDA. Supporting unbiased decision-making, free of undue influence from funders or other HIC stakeholders, was seen as critical for enabling local ethics assessment. Participants from LMICs placed greater value on this local perspective (e.g., members of a local REC we interviewed placed high value on a trusted local investigator’s assessment of an MDA study).

Third, we observed the importance of thoughtful RECs and DSMCs in bringing ethical issues to the fore. RECs and DSMCs need not be focused on only direct risks and benefits (e.g., adverse drug events). In one instance we observed, the DSMC was empowered with local voices and saw its role as bringing up longer-term community risks and harms of MDA, from AMR to unintended burdens on communities and local health systems where MDA occurs.

A fourth and unexpected finding was the sometimes overlooked role of community health workers (CHWs) in MDA. While not traditional research participants, some asked whether RECs needed to protect CHWs as research participants (if they are exposed to risks of AMR, by virtue of close participant contact) or whether funders and investigators should do more to protect CHWs.

Together, our findings suggest that approaching MDA research as if it were simply another clinical trial fails to appreciate fully the ethics of MDA. Contemporary MDA studies are large, sometimes involving tens of thousands of individuals (or more), causing some to ask if this were de facto implementation ahead of the evidence. Likewise, the desire to partner MDA with existing public health interventions (e.g., vaccine campaigns or seasonal malaria chemoprophylaxis) or via the local health ministry, while motivated by concern for efficiency and avoiding duplicative efforts or burdens on the health system, could inadvertently promote a belief that MDA of azithromycin to reduce childhood mortality is a proven public health intervention and complicate informed consent. That AMR could persist beyond the study period and affect communities who did not directly participate makes MDA a social concern beyond any single study.

Ethical concerns about an ethical double standard (our unifying theme) are not new in global health. They were expressed regarding placebo-controlled HIV studies in the 1990s [42–45] and more recently over the use of less-expensive, less-effective medicines globally [46]. On the one hand, participants expressed that it is a double standard to use MDA in LMICs (e.g., because HICs would not tolerate MDA, instead addressing root causes of child mortality, or because typically HICs do not experience the child mortality rates that justify MDA). On the other hand, some considered it a double standard not to use MDA in LMICs (because communities in HICs use antibiotics in higher amounts, for less severe clinical conditions, such as acne). Although high antibiotic use in HICs does not justify using MDA in LMICs [19], one can appreciate the dilemma.

Our participants’ concerns over a double standard at times related to the contemporary attention being given to “decolonizing” global health. Some invoked the idea directly, suggesting people in HICs demonstrate a colonial-style mindset by being more fearful of risks affecting them (e.g., global AMR) than about MDA’s benefits for others [21]. As one said, “…this is like imperialism again. We know that this medicine given to kids prophylactically in Africa saves lives. But against the wildly low likelihood of a superbug developing, we don't think you should do it. Bollocks.”

By contrast, others saw evidence of neocolonialism in funders, investigators, and RECs from HICs, whom they perceived as privileging their own interests in short-term “magic bullets” rather than longer-term investments in community-based health programs. This sentiment echoes the idea that the shadow cast by colonialism encourages favoring “superficial” fixes to global health programs rather than structural, longer-term solutions [47].

Decolonizing global health is a contested concept in HICs and LMICs alike, with some concern that the concept itself has been misappropriated and misused by people in HICs in order to support their own interests or allay feelings of guilt [48–50]. To the extent that the contemporary movement focuses on eliminating neocolonial influences (e.g., financial, structural, and cultural) and power imbalances that interfere with local, autonomous decision-making, it joins decades of work by civil society, activists, and others who have worked tirelessly to equalize power and achieve global health equity.

Situating MDA within a neocolonial context will not explain or solve all the ethical challenges MDA raises, but it can be analytically useful. It should help encourage critical evaluation of the privileging of some individuals’ or institutions’ interests and decisions over others’. It reminds us that MDA is part of the broader challenge of equalizing power and promoting global health equity. It should cause us to ask different questions. By analogy, in responding to bioethicists’ arguments supporting the use of less-effective, less-expensive medicines globally, some criticized this as endorsing an “ethics of resignation” whereby “resource scarcity is accepted as inevitable” [51]. We ought not see the ethics of MDA as fixed within the current *status quo* as an isolated problem to be solved; instead, we must ever consider the structural factors that create the need for MDA in the first place.

Our study has two main implications. First, our participants expressed clearly the need for additional research into both the effectiveness and longer-term effects of azithromycin MDA, particularly the need to understand the mechanism of MDA for childhood mortality, to gauge the magnitude of AMR, and to determine the connection, if any, between AMR and real clinical outcomes (e.g., by monitoring for resistant infections near where MDA occurs). Although anti-parasitic MDA for malaria is not perfectly analogous to MDA using antibacterials, some of the lessons learned to mitigate the risk of malaria resistance—from effectively targeting local disease patterns with MDA and alternative interventions to ensuring both social and political support—could inform priority research areas for MDA [52–54].

Second, there is a need to develop more comprehensive ethics guidance and frameworks for azithromycin MDA—guidance that emphasizes ethical obligations beyond regulatory compliance; engagement and empowerment of not only communities but a range of stakeholders, from funders to national and international regulatory authorities; a primary emphasis on overall social value to the long-term global health agenda; and open public discussion of these ethical issues [55–57]. There is unlikely to be a one-size-fits-all ethics approach. One of the challenges is that we do not seem to have an ethical vantage point from which MDA might be judged as unequivocally fair. As a result, differences persist in stakeholder views about how we should: weigh overuse of azithromycin in HICs versus faulting LMICs for azithromycin use in MDA; consider a “do no harm” approach versus one emphasizing net clinical or societal benefits; evaluate competing interventions and opportunity costs; and understand MDA through the lens of global health colonialism versus stakeholder self-interest. In 2020, the WHO suggested the need to revisit its guidance on azithromycin MDA in 2–3 years. When this time comes, we recommend a dedicated effort to integrate comprehensive analyses of current ethics and policy issues into this guidance. Any ethics framework created must be narrow enough to address the most pressing challenges for azithromycin MDA and broad enough to accommodate the likelihood that other antibiotics may be proposed for MDA and that any guidance must be sensitive to local contextual factors related to antibiotic use.

Like all studies, ours has limitations. Although we were able to elicit the perspectives from diverse global stakeholders associated with a number of important MDA studies, we did not comprehensively document perspectives from all stakeholders in all MDA studies. We interviewed disproportionately clinical investigators, and then sampled based on to whom they referred us. Those actively engaged in, or hoping for future, funded work might have observations influenced by this or limited by what they felt comfortable sharing. We had no independent check of some assertions, though this limitation was partly mitigated by this study’s role in a larger project where we had perspectives from commissioned expert papers and expert consultation discussions. Also, while we aimed to have substantial representation from participants from LMICs, language barriers and connection problems created challenges for interpreting these interviews. Finally, qualitative research involves an element of subjectivity. Although as researchers we engaged in techniques, such as reflexivity (i.e., acknowledging our own biases), we acknowledge that different researchers interviewing the same or different participants might come to a different understanding of our phenomenon of interest, the ethical challenges of MDA.

## Conclusions

Our study of the real-world perspectives regarding ethics and the use of antibiotic MDA for childhood mortality suggests that its fundamental ethics are far from settled. Situating MDA within broader issues of standards, equity, and power in global health can broaden and deepen our understanding of the ethical challenges of MDA of antibiotics to reduce childhood mortality. To develop more robust ethics guidance for MDA, there is an urgent need for data to evaluate, at a granular level, the risks and benefits of MDA in specific communities with concurrent ethics analysis to understand its underlying assumptions and implications.

## Data Availability

Deidentified data are available from the authors upon reasonable request.

## Abbreviations

AMR: Antimicrobial resistance
CHW: Community health workers
DSMC: Data safety and monitoring committee
HIC: High-income country
IRB: Institutional review board
LMIC: Low- or middle-income country
MORDOR: Macrolides Oraux pour Réduire les Décès avec un Oeil sur la Résistance
MDA: Mass drug administration
REC: Research ethics committee
WASH: Water, sanitation, and hygiene
WHO: World Health Organization
